# Challenges for sustainability of the open access model: Brazilian health journals

**DOI:** 10.1590/1518-8345.0000.2827

**Published:** 2016-12-08

**Authors:** Lilian Nassi-Calò

**Affiliations:** Coordinator of Scientific Communication in Health at BIREME/PAHO/WHO and a collaborator of SciELO. Email: calolili@paho.org

**Figure:**
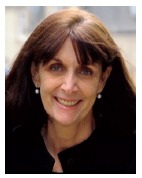

The open access movement initiated in the late 1990s aimed to eliminate economic and copyrights barriers in accessing and disseminating knowledge. Motivated by the abusive increase in journals subscriptions by international publishers who dominated - and still do so to some extent - the global publishing market, open access (OA) is made feasible through the World Wide Web and information and communication technologies. 

This business model has been asserting itself throughout the world as the preferred form of publication of research results, especially those financed with public resources. Its widespread adoption, however, has not yet been fully achieved because researchers are not entirely sure that the reward and evaluation mechanisms of science recognize this modality at the same level as mainstream subscription journals.

OA has been consolidated through successive mandates from institutions, funding agencies and governments around the world. Today, it is estimated that between 35 and 60% of peer-reviewed articles - a number that increases by 2% a year - are published in open access, depending on the platform where they are indexed^1^. This percentage considers only the Gold Route (OA journals); postprint repositories (Green Route), and more recently, preprint repositories^2^ add to journals, making most of science openly available not only to academia, but to all sectors of society. There is also another model, the hybrid journals, which are subscription publications that at the authors' option and payment of a fee make the articles available in OA. The benefits of equitable access to scientific and technical information - not only in developing countries - are evident and undeniable, since they contribute to education, continuing professional development, and the nations' technological and economic growth.

OA has drastically altered the paradigm of scientific publications leading to the elimination of the print version of most journals. The costs were, thus, greatly reduced, enabling the emergence of journals supported by scientific societies, research institutions and funding agencies, such as the SciELO Program created in 1998, financed mainly by FAPESP. SciELO offers a platform that creates scale for indexing, publishing and interoperability of journals, always following the state-of-the-art of publishing methodologies and technologies. Therefore, SciELO provides the journals a common saving of resources while minimizing costs and maximizing their visibility, impact, and international presence. Next came up BioMed Central, the first OA commercial publisher, and the Public Library of Science, which started to require an article processing charge (APC) to make articles freely available to readers. 

According to the Directory of Open Access Journals (DOAJ)^3^, which only includes fully OA journals, only 26% of the 9,192 journals levy a publication fee. Of the 94 health journals in SciELO Brazil, only 17 journals (18%) use this source of income^4^. Although a small percentage of journals levy a publication fee, the amounts vary widely. In the case of SciELO Brazil, it oscillates between US$ 45 and US$ 910. These figures are well below those of megajournals such as PLoS One (US$ 1,495), PLoS Biology and PLoS Medicine (both US$ 2,900).

Studies show that the resources employed by libraries to pay for journal subscriptions would be more than sufficient to finance the open access model through APCs^5^. While this transition is not made, many funding agencies have allowed researchers to include the payment of APCs in their research projects, understanding that the publication of research outputs is the final step of these projects. Thus, it is unusual for researchers or graduate students to take personal responsibility for their payment.

The scenario of OA publishing today differs from that projected at the beginning of the movement. The initial perspective was to finance publication fees to favor the business model, which would go down "and will continue to do so, asymptotically approaching zero" in the conception of PLoS co-founder Michael Eisen^6^. This was not the case, since APCs of the aforementioned PLoS Medicine and PLoS Biology practically doubled between 2009 and 2012. In addition, the appearance of predatory journals and publishers^7^ ^*^ is worrisome for two main reasons: the first concerns the risk of well-meaning researchers sending their manuscripts to seemingly legitimate journals and accepting invitations to join their editorial boards; the second is the threat that this fraudulent practice imposes on open access, causing many to confuse both concepts and envisage all open access publications as of poor quality, flawed and lacking peer review, aiming only to collect APCs. All predatory journals are OA, but the reciprocal is not true, and the scientific community must be alerted, far beyond what claims Jeffrey Beall's list^8^ and his unequivocal intention to denigrate open access as a whole.

The future and sustainability of OA was the theme of the recent workshop AlterOA^9^ ^)^ organized by the European Commission in 2015. The meeting showed that innovative ideas and technologies are key to ensuring the sustainability of OA globally. Partnerships between academia, editors, libraries, and publishers allow creating non-commercial initiatives for OA publishing, such as the Open Research Network, Open Library of Humanities, and Horizon2020. Coupling OA publishing and research data sharing (open data) strengthens this business model, enables its preservation and promotes wide use and citation of data, maximizing the return of research investment^10^.

The scenario of open access publishing in Brazil and Latin America is one of the most favorable worldwide. The characteristics of its business models - small non-for-profit publishers funded by educational and research institutions and funding agencies not only ensure the region's very low index of predatory journals, but also enable countries in the region to adopt the fastest growing form of publication in the world. The SciELO Program, since 1998, has played a major role in promoting the integrated governance of journals of the collection and facilitating open access. Subbiah Arunachalam, a longtime advocate of the OA movement, stresses the role of SciELO in the region by stating that "with these efforts, Latin America has become a model for affordable OA journal publishing" and believes that when India and China adopt this model "that would have a great impact on making science open, not only in these regions but around the world"^11^.

## Footnotes

* In academic publishing, predatory open access publishing is an exploitative open-access publishing business model that involves charging publication fees to authors without providing the editorial and publishing services associated with legitimate journals (open access or not).
